# Forced expression of the cell cycle inhibitor p57^Kip2 ^in cardiomyocytes attenuates ischemia-reperfusion injury in the mouse heart

**DOI:** 10.1186/1472-6793-8-4

**Published:** 2008-02-29

**Authors:** Sheila A Haley, Ting Zhao, Lijun Zou, Jan E Klysik, James F Padbury, Lazaros K Kochilas

**Affiliations:** 1 Department of Molecular Biology, Cell Biology and Biochemistry, Brown University, Providence, RI 02912, USA; 2 Department of Pediatrics, Division of Neonatal-Perinatal Medicine, Women and Infants Hospital, Providence, RI 02903, USA; 3 Department of Pediatrics, Division of Pediatric Cardiology, Rhode Island Hospital, Providence, RI 02903, USA; 4 Department of Pediatrics, Warren Alpert School of Medicine, Brown University, Providence, RI 02912, USA

## Abstract

**Background:**

Myocardial hypoxic-ischemic injury is the cause of significant morbidity and mortality worldwide. The cardiomyocyte response to hypoxic-ischemic injury is known to include changes in cell cycle regulators. The cyclin-dependent kinase inhibitor *p57*^*Kip*2 ^is involved in cell cycle control, differentiation, stress signaling and apoptosis. In contrast to other cyclin-dependent kinase inhibitors, p57^Kip2 ^expression diminishes during postnatal life and is reactivated in the adult heart under conditions of cardiac stress. Overexpression of *p57*^*Kip*2 ^has been previously shown to prevent apoptotic cell death *in vitro *by inhibiting stress-activated kinases. Therefore, we hypothesized that *p57*^*Kip*2 ^has a protective role in cardiomyocytes under hypoxic conditions. To investigate this hypothesis, we created a transgenic mouse (*R26loxpTA-p57*^*k*/+^) that expresses p57^Kip2 ^specifically in cardiac tissue under the ventricular cardiomyocyte promoter *Mlc2v*.

**Results:**

Transgenic mice with cardiac specific overexpression of *p57*^*Kip*2 ^are viable, fertile and normally active and their hearts are morphologically indistinguishable from the control hearts and have similar heart weight/body weight ratio. The baseline functional parameters, including left ventricular systolic pressure (LVSP), left ventricular end diastolic pressure (LVEDP), LVdp/dt_max_, heart rate (HR) and rate pressure product (RPR) were not significantly different between the different groups as assessed by the Langendorff perfused heart preparation. However, after subjecting the heart *ex vivo *to 30 minutes of ischemia-reperfusion injury, the *p57*^*Kip*2 ^overexpressing hearts demonstrated preserved cardiac function compared to control mice with higher left ventricular developed pressure (63 ± 15 vs 30 ± 6 mmHg, p = 0.05), rate pressure product (22.8 ± 4.86 vs 10.4 ± 2.1 × 10^3^bpm × mmHg, p < 0.05) and coronary flow (3.5 ± 0.5 vs 2.38 ± 0.24 ml/min, p <0.05).

**Conclusion:**

These data suggest that forced cardiac expression of p57^Kip2 ^does not affect myocardial growth, differentiation and baseline function but attenuates injury from ischemia-reperfusion in the adult mouse heart.

## Background

Ischemic heart disease is the leading cause of morbidity and mortality in the industrialized world, but the development of effective therapy has been hampered by the lack of mechanistic insights into the physiological response of the heart to hypoxic stress. Adult cardiomyocytes respond to hypoxic stress by reverting to genetic programs associated with embryonic cardiac development, collectively referred to as "the fetal gene program" [1]. One of the most critical factors controlling heart growth and development is intrauterine oxygen availability [2,3]. Embryonic cardiac development occurs in an environment of low oxygen tension and hypoxia regulates several stress-related pathways affecting cellular proliferation, differentiation and death. While the low oxygen tension at this stage of development is "physiologic", the genes whose expression is modulated by differences in oxygen tension are adapted to the ambient levels of oxygen during this period. Recapitulation of this developmental program may be a physiologic adaptation that allows the ischemic heart to respond to hypoxia.

Heart cells divide during development and then exit the cell cycle as they undergo terminal differentiation [4,5]. This lack of proliferative capacity poses a challenge for hearts that undergo ischemic injury as they loose cardiac mass and terminal heart failure frequently results. During development, cardiomyocytes proliferate extensively. Cells are driven through the cell cycle by a complex of two classes of proteins, cyclins and cyclin dependent kinases (CDKs). This complex is regulated by two families of cyclin-dependent kinase inhibitors (CKIs). The INK4 family (p16^Ink4a^, p15^Ink4b^, p18^Ink4c^, p19^ARF^) specifically inhibit CDK4 and CDK6, while the CIP/KIP family (p21^Cip1^, p27^Kip1^, and p57^Kip2^) inhibit all G1/S CDKs [6,7]. The CKIs are implicated in cell cycle exit and terminal differentiation in a number of cell types [8-10]. Of all the CKIs, only p57^Kip2 ^has been shown to be essential for embryonic development, as *p57*^Kip2 ^null mice display significant congenital defects such as cleft palate, omphalocele and short limbs [11-13]. In addition, the *p57*^Kip2-/- ^mice exhibit increased apoptosis in many tissues, including the heart. They typically die *in utero *or soon after birth due to aspiration and respiratory insufficiency from the cleft palate [12,13]. In the mouse heart, *p57*^Kip2 ^message is up-regulated during mid-gestation [12,14]. The p57^Kip2 ^protein levels peak during late gestation, and then disappear during early fetal life [15]. Low levels of p57^Kip2 ^protein are present in the adult human heart, which then increase during terminal heart failure [16]. By contrast, there is a concomitant decrease in p21^Cip1 ^and p27^Kip1 ^levels [16].

Apart from inhibiting the cell cycle, p57^Kip2 ^has additional roles in the cell. For example, p57^Kip2 ^modulates stress activated signaling by functioning as an endogenous inhibitor of c-Jun kinase (JNK/SAPK), as the QT domain of p57^Kip2 ^binds and inhibits the JNK/SAPK pro-apoptotic activity [17]. In addition, *p57*^*Kip*2 ^is up-regulated at early time points of hypoxia [18], indicating it is within the early wave of hypoxia-responsive genes rather than being secondarily induced. Previous studies have demonstrated that p21^Cip1 ^and p27^Kip1 ^overexpression in cultured rat cardiomyocytes protects the cells from hypoxia-induced apoptosis, and this protection appears to be independent of CKI activity [19]. Because p57^Kip2 ^is differentially regulated from the two other CIP/KIP family members during hypoxic stress, it is plausible that it possesses similar cardioprotective properties in this setting and compensates for the observed downregulation of p21^Cip1 ^and p27^Kip1 ^in the stressed heart [20]. The possibility that p57^Kip2 ^has a previously unrecognized role in cardiac biology related to protection from hypoxic-ischemic injury has not been examined. We hypothesized that p57^Kip2 ^protects cardiomyocytes under conditions of limited oxygen supply as occurs during embryonic cardiac development and in ischemic injury of the adult heart.

To evaluate this hypothesis, we generated a mouse model (*R26loxpTA-p57*^*k*/+^) that allows cre-inducible functional expression of *p57*^*Kip*2 ^in a tissue-specific fashion. We used this transgenic model in combination with the *Mlc2v- Cre*^*k*/+ ^transgenic mouse to force myocardial specific *p57*^*Kip*2 ^expression in the embryonic and adult heart [21] and we show that myocardial specific expression of *p57*^*Kip*2 ^attenuates hypoxic-ischemic injury in the adult mouse heart. These findings suggest that p57^Kip2 ^may represent a developmentally regulated protein aiming to protect cardiomyocytes under conditions of limited oxygen supply during development and in pathologic ischemic conditions of adulthood.

## Results

### Cardiac specific overexpression of p57^Kip2 ^does not affect heart development or cardiomyocyte proliferation

**Figure 1:**
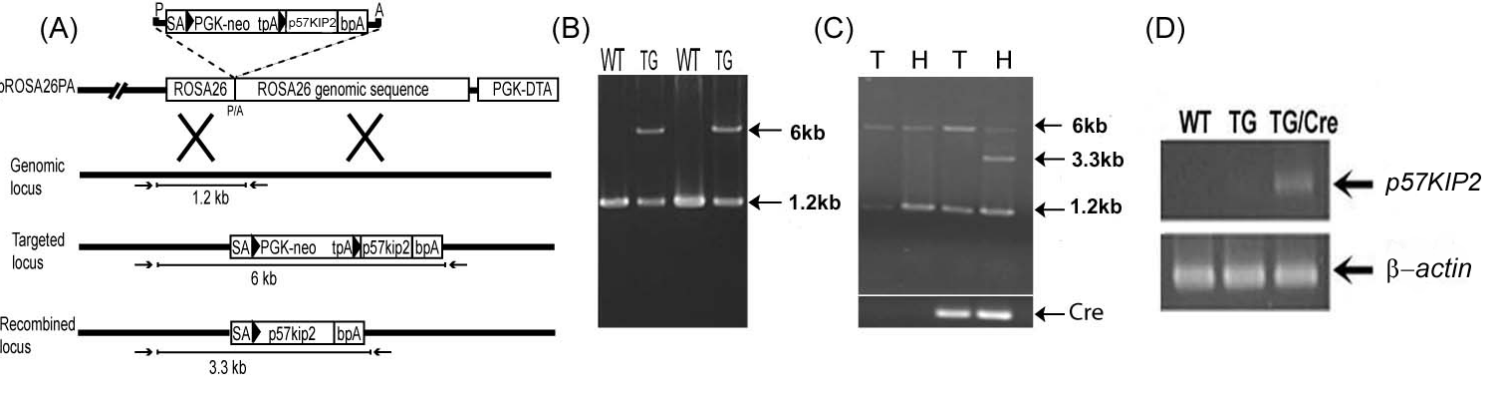
**Creation of a transgenic mouse with forced expression of p57^Kip2 ^in a cre dependent manner**. **(A) **Recombination scheme displaying the structure of the targeting vector, genomic R26 locus, targeted locus after homologous recombination and recombined locus after *cre *activation **(B) **PCR genotype analysis of tail genomic DNA (gDNA) **(C) **PCR analysis shows that *cre-*induced recombination is restricted to the heart, and occurs only in the presence of cre recombinase. T= tail gDNA, H = heart gDNA **(D) **RT-PCR analysis demonstrates robust expression of p57^Kip2 ^message in adult transgenic hearts in the presence of *cre *recombinase, compared to barely detectable levels in the absence of *cre *recombinase.

Since the *p57*^*Kip*2 ^cDNA is preceded by a loxP-flanked strong transcriptional termination sequence (*tpA*), in the absence of cre-recombinase *p57*^*Kip*2 ^transcription is terminated prematurely and the generated transgenic mice (*R26loxpTA-p57*^*k*/+^) were phenotypically normal as expected. When these mice were crossed with the *Mlc2v- Cre*^*k*/+ ^transgenic mice that express cre-recombinase under the transcriptional control of the myosin light chain-2 ventricular (*mlc2v*) promoter, the cre-mediated excision of the floxed termination sequence led to forced expression of *p57*^*Kip*2 ^in ventricular cardiomyocytes. Fifty-three double heterozygous animals (*R26loxpTA-p57*^*k*/+^*/Mlc2v-Cre*^*k*/+^*) *from these crosses have been analyzed. The double transgenic mice developed normally and no defects in embryos or adults were observed. Litter sizes and fertility were similar to those of control mice and offspring were produced in the expected Mendelian ratios (Table 1). None of the *R26loxpTA-p57*^*k*/+^*/Mlc2v-Cre*^*k*/+ ^mice were prone to early lethality over 2 years of observation suggesting that cardiac specific overexpression of *p57*^*Kip*2 ^is well tolerated from the very early stages of myocardial differentiation. Ventricular tissue-specific cre-recombination that allows *p57*^*Kip*2 ^over-expression in cardiomyocytes of the compound heterozygous offspring could be detected by PCR as shown in the diagram (Figure 1A, 1C).

**Table 1:** Genotype analysis of offspring resulting from *R26loxp-TAp57*^*k*/+^*;Mlc2v-cre*^*k*/+^crosses.

	**Genotype**
	n = 203
***R26loxp-TAp57*^*k*/*k*^*;Mlc2v-cre*^*k*/+^**	13
***R26loxp-TAp57*^*k*/*k*^*;Mlc2v-cre*^+/+^**	21
***R26loxp-TAp57*^*k*/+^*;Mlc2v-cre*^*k*/+^**	53
***R26loxp-TAp57*^*k*/+^*;Mlc2v-cre*^+/+^**	50
***R26loxp-TAp57*^+/+^*;Mlc2v-cre*^*k*/+^**	25
***R26loxp-TAp57*^+/+^*;Mlc2v-cre*^+/+ ^(WT)**	41

The *R26loxp-TAp57*^*k*/+^*;Mlc2v-cre*^*k*/+ ^genotype is represented in the expected Mendelian ratio in the offspring. However, mice with p57^KIP2 ^cardiac-specific overexpression that are homozygous for the *R26p57 *allele (*R26loxp-TAp57*^*k*/*k*^*;Mlc2v-cre*^*k*/+^) are significantly under-represented in the offspring (p < 0.01 by Chi-square and n = 203)

**Figure 2:**
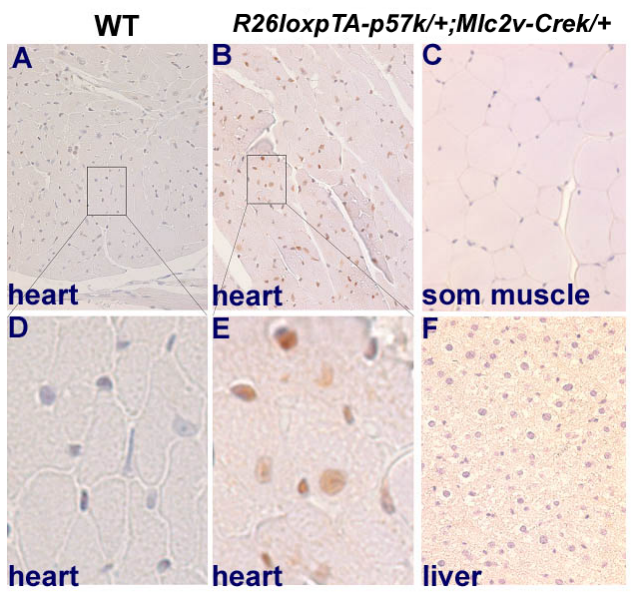
**p57^Kip2 ^protein is expressed specifically in the adult transgenic mouse heart**. p57^Kip2 ^expressed in the hearts of adult *R26loxpTA-p57*^*k*/+^*;Mlc2v-Cre*^+/- ^mice **(B, E)**, while it is absent from WT hearts **(A, D)**. p57^Kip2 ^expression is restricted to the hearts of transgenic animals and is absent from adult muscle **(C) **and liver **(F)**.

We examined p57^Kip2 ^expression by reverse transcriptase (RT-PCR) and immunohistochemistry in wild type (WT) or single (*R26loxpTA-p57*^*k*/+^) transgenic hearts and compared them to the adult double transgenic (*R26loxpTA-p57*^*k*/+^*/Mlc2v-Cre*^*k*/+^*) *hearts. RT-PCR analysis demonstrated the presence of *p57*^*Kip*2 ^message in the adult double transgenic heart, while at the same number of cycles, there was no visible band in the adult WT or single transgenic heart (Figure 1D). The average *p57*^*Kip*2 ^expression by quantitative RT-PCR analysis was found to be 2.7 fold higher in compound transgenic hearts (data not shown). Taking into consideration that only 14–20% of adult mouse ventricular cells are cardiomyocytes and the reported success of recombination is approximately 80% with the *Mlc2v-Cre *mouse [21], this result indicates that the cardiomyocytes of the compound transgenic mice express an 8–12 fold higher level of *p57*^*Kip*2 ^transcripts above the wild type control animals [5,21]. Since there may still be some non-ventricular tissue in this preparation, this number represents a lower estimate of the efficiency of our transgenic system at the mRNA level. Immunohistochemistry demonstrated that p57^Kip2 ^is abundantly present in the nuclei of adult cardiomyocytes in the *R26loxpTA-p57*^*k*/+^*;Mlc2v-Cre*^*k*/+ ^transgenic mice (approximately 80% of the cardiomyocytes nuclei are stained for p57^Kip2^, whereas it is virtually absent from the cardiomyocytes of wild-type animals (Figure 2). We did not observe p57^Kip2 ^expression in the skeletal muscle or in the liver of the double transgenic animals (Figure 2F, 2G). These results indicate that the transgene is not only specific and effective in overexpressing p57^Kip2 ^in cardiomyocytes, but also that the cellular capacity for degradation of p57^Kip2 ^is overcome and not sufficient to reduce the elevated protein levels to normal.

**Figure 3:**
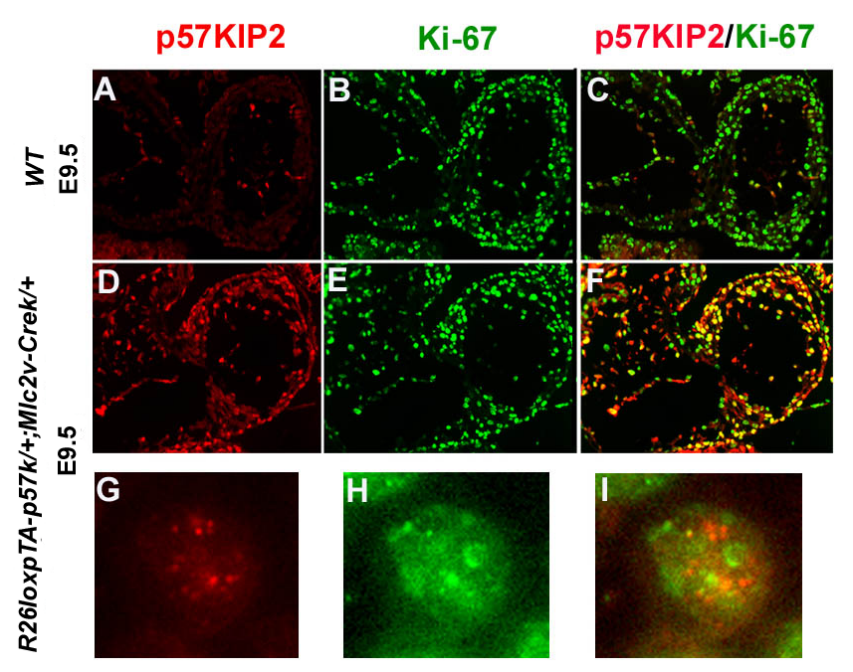
**Forced *p57*^*Kip*2 ^expression in the embryonic heart does not impair cardiomyocyte proliferation**. Localization of p57^Kip2 ^and Ki-67 in ventricular cardiomyocytes of E9.5 wild type (WT) and *R26loxpTA-p57*^*k*/+^*;Mlc2v-Cre*^*k*/+ ^animals by indirect immunofluorescence. In WT E9.5 cardiomyocytes p57^Kip2 ^is not yet expressed **(A-C)**. However, p57^Kip2 ^is expressed in most cardiomyocytes of the *R26loxpTA-p57*^*k*/+^*;Mlc2v-Cre*^+/- ^mouse embryos as early as E9.5 **(D-F)**. Expression of p57^Kip2 ^in embryonic cardiomyocytes does not preclude Ki-67 expression **(E, F)**. DNA synthesis in the heart of *R26loxpTA-p57*^*k*/+^*;Mlc2v-Cre*^*k*/+ ^transgenic mice is similar to the hearts of wild type animals despite the abundance of p57^Kip2 ^protein in the cardiomyocytes of the transgenic hearts. In the merged pictures (third column), the yellow nuclei represent cardiomyocytes co-labeled by both antibodies. High power magnification of the same cardiomyocyte labeled with both antibodies, illustrates that although there are many cells stained with both p57^Kip2 ^and Ki-67, in most cells p57^Kip2 ^and Ki-67 immunoreactivity occur in exclusive nuclear domains **(G, H, I)**.

During development, *p57*^*Kip*2 ^gene expression is first observed in the heart of WT mice at E10.5 [14,15]. By E11.5, p57^Kip2 ^protein is present in the nuclei of the endocardial cells and in about 75% of the WT cardiomyocytes (Figure 3). In the double transgenic embryos, p57^Kip2 ^was first detected in cardiomyocyte nuclei at E9.5, following the expression of *Mlc2v-Cre *during early myocardial differentiation [22], indicating that p57^Kip2 ^expression is directed by the *Mlc2v *promoter (Figure 3D).

Gross cardiac defects and histological evidence of ventricular thinning, hypertrophy, or fibrosis were absent in adult p57^Kip2 ^overexpressing hearts. Similarly, the ratio of heart weight to body weight (HW/BW) of the transgenic hearts did not significantly differ from that of wild type animals (6.6 × 10^-3 ^± 0.2 × 10^-3^, m ± SEM, n = 8 vs. 7.5 × 10^-3 ^± 0.7 × 10^-3^, n = 4) at 3 months of age. There was also no difference in the rate of apoptosis at this age as determined by TUNEL assay (data not shown). To assess whether cardiomyocyte-specific *p57*^*Kip*2 ^overexpression affects cardiomyocyte proliferation we performed immunofluorescence experiments with Ki-67 antibody, a marker for DNA synthesis and cell cycling in E8.5-E11.5 hearts [23]. We did not detect any difference in the number of Ki-67 positive cardiomyocytes between p57^Kip2 ^overexpressing transgenic and control animals (Figure 3). These results indicate that p57^Kip2 ^overexpression does not affect the ability of cardiomyocytes to enter S-phase. Interestingly, in some of the cells, p57^Kip2 ^and Ki-67 immunoreactivity was present in the same nucleus, although they appeared in mutually exclusive nuclear areas (Figure 3G–I). Such cells were found in both transgenic and control animals and therefore cannot be attributed to a transgene effect. This is in accordance with the pattern of p57^Kip2 ^expression described in trophoblast giant cells, in which BrdU and p57^Kip2 ^co-immunoreactivity could be detected after the cells committed to endoreduplication [24]. Endoreduplication is a process of repeated rounds of DNA synthesis in the absence of mitosis, which occurs in both trophoblast cells and cardiomyocytes and results in the formation of polyploid cells. Thus, it is possible that in cardiomyocytes, p57^Kip2 ^expression and terminal differentiation are not associated with cell cycle exit but rather with endoreduplication.

### p57^Kip2 ^expression in adult cardiomyocytes protects hearts from ischemia/reperfusion injury

**Figure 4:**
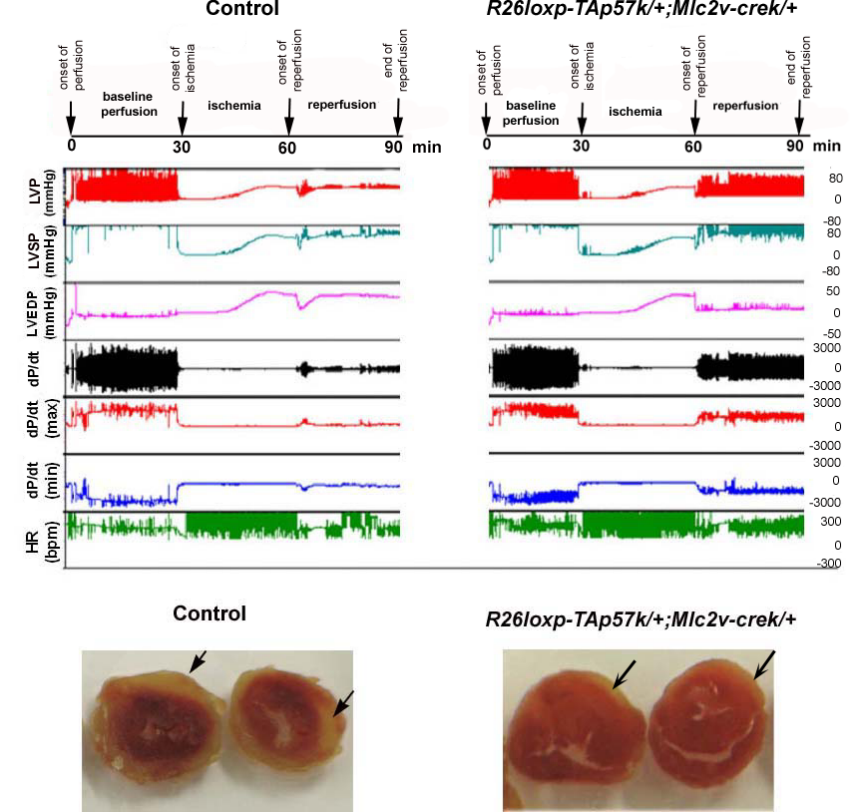
**p57^Kip2 ^protects hearts from ischemia/reperfusion (I/R) injury in the Langendorff isolated perfused heart model**. Upper Panel: Representative hemodynamic tracings from Langendorff isolated perfused heart from a control and a *R26loxpTA-p57*^*k*/+^*;Mlc2v-Cre*^*k*/+ ^animal. Real-time condensed recordings of hemodynamic parameters (LVP=left ventricular pressure, LVSP=left ventricular systolic pressure, LVEDP=left ventricular end diastolic pressure, dp/dt_(max/min) _and HR) are shown. Lower Panel: Representative sections of a control (left) and a R26loxpTA-p57^*k*/+^;Mlc-2v^+/- ^(right) heart. The hearts were sliced into sections and stained with 2,3,5-TTC followed by formalin fixation. Viable tissue is stained brick red, while infarcted areas are white. Note the more significant necrotic area (black arrow) in the injured, control heart.

**Figure 5:**
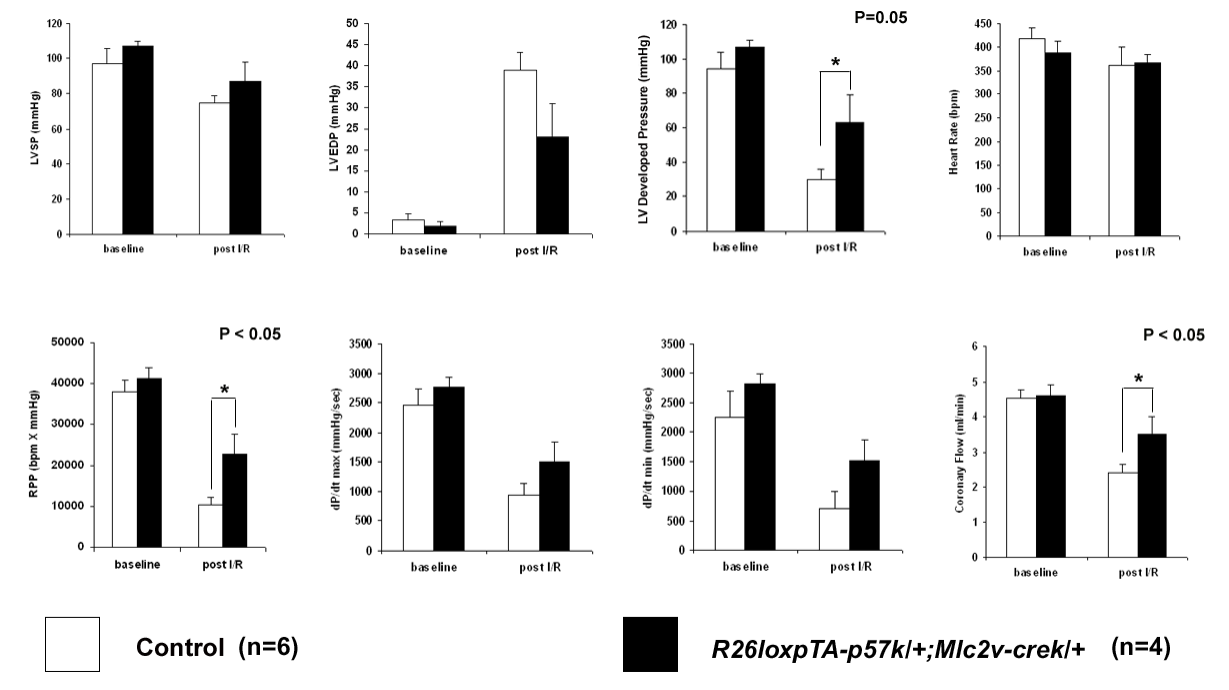
**Summary of hemodynamic data from the isolated heart perfusion evaluation of control and p57^Kip2 ^overexpressing mice (*R26loxpTA-p57*^*k*/+^*;Mlc-2vCre*^*k*/+^), at baseline and post ischemia/reperfusion (I/R)**. Left Ventricular Systolic Pressure (LVSP), Left Ventricular End Diastolic Pressure (LVEDP), Left Ventricular Developed Pressure (LVDP), Heart Rate (HR), Rate Pressure Product (RPP), LVdp/dt_(max/min) _and Coronary Flow (CF). LVDP, RPP and CF were better conserved in the transgenic hearts to a statistical significant degree compared to the hearts of wild type littermates (p < 0.05).

After we characterized histologically the adult transgenic mice and demonstrated no differences in 3 months of age, we proceeded with the analysis of cardiovascular function. We studied age matched adult mice from the same cohort that covered the adult lifespan from 14–30 weeks, when the excitation-coupling mechanisms are known to remain constant [25]. To assess the contractile function of *p57*^*Kip*2 ^overexpressing transgenic hearts, we utilized the Langendorff isolated perfused mouse heart preparation as previously described [26-28]. After 30 minutes of stabilization baseline functional data were obtained and the hearts were then subjected to a protocol of 30 minutes global ischemia followed by 30 minutes reperfusion. This protocol is based on our experience from previous studies [26-28]. In one of the experiments we assessed the myocardial necrotic area after staining with 10% 2,3,5-triphenyltetrazolium chloride (TTC) and we found that the area of necrotic tissue was reduced (20% vs. 37%) in the double transgenic heart compared with the control (Figure 4). The baseline functional parameters, including left ventricular systolic pressure (LVSP), left ventricular end diastolic pressure (LVEDP), LVdp/dt, rate pressure product (RPP) and heart rate (HR) were not significantly different between the different groups. However, after subjecting the hearts to 30 minutes of ischemia, the reperfused *p57*^*Kip*2 ^expressing hearts exhibited significantly better preservation of cardiac function than the WT hearts. More specifically the transgenic hearts demonstrated higher left ventricular developed pressure (LVDP), rate pressure product (RPP) and coronary flow (CF) as assessed at the end of the reperfusion period (p ≤ 0.05, Figure 5; Table 2). The hemodynamic benefits were apparent from the onset of the reperfusion stage and were maintained throughout the 30 min period, suggesting that the protective effect of the p57^Kip2 ^transgene may be manifest during the acute hypoxic phase and not restricted to the reperfusion phase of the injury.

**Table 2:** Description of experimental animals and cardiac functional parameters

	**Control**	***R26loxp-TAp57***^*k*/+^***; Mlc2v-cre***^*k*/+^
	n = 6	n = 4
Age (days old)	136 ± 22	133 ± 23
Body Weight (grams)	32 ± 1	34.2 ± 1
Heart Weight (mg)	162 ± 2	214 ± 2
Heart/Body Weight Ratio (mg/g)	5.03 ± 0.45	6.19 ± 0.75
**Baseline functional parameters**		
LVSP (mmHg)	97 ± 9	107 ± 3
LVEDP (mmHg)	3.4 ± 1.4	2 ± 1
LVDP (mmHg)	94 ± 10	105 ± 3
HR (bpm)	418 ± 22	387 ± 24
RPP (bpm × mmHg)	37,920 ± 3,064	41,218 ± 2,794
dP/dt max (mmHg/sec)	2,461 ± 262	2,764 ± 180
CF (ml/min)	4.53 ± 0.24	4.58 ± 0.26
**Functional Parameters post I/R**		
LVSP (mmHg)	75 ± 3.5	87 ± 11
LVEDP (mmHg)	39 ± 4.3	23 ± 8
LVDP (mmHg)	30 ± 6*	63 ± 15*
HR (bpm)	362 ± 37	368 ± 17
RPP (bpm × mmHg)	10,361 ± 2,077**	22,787 ± 4,858**
dP/dt max (mmHg/sec)	944 ± 193	1,513 ± 332
CF (ml/min)	2.38 ± 0.24**	3.5 ± 0.5**

Values are means ± SE. The two asterisks (**) indicate statistical significance with a probability of p < 0.05 (unpaired *t-test*), while the one asterisk indicates a probability of p = 0.05. One-way ANOVA followed by *Fisher post hoc *test also indicated significant difference among these groups. There was also a strong trend for lower LVEDP in the transgenic hearts; however with the small number of tested animals this did not reach statistical significance. LVSP, left ventricular systolic pressure; LVEDP, left ventricular end diastolic pressure; LVDP, left ventricular developed pressure, HR, heart rate; RPP, rate pressure product; CF, coronary flow.

### p57^Kip2^-mediated cardioprotection in the *ex vivo *ischemia reperfusion injury is associated with modulation of the stress signaling cascade

**Figure 6:**
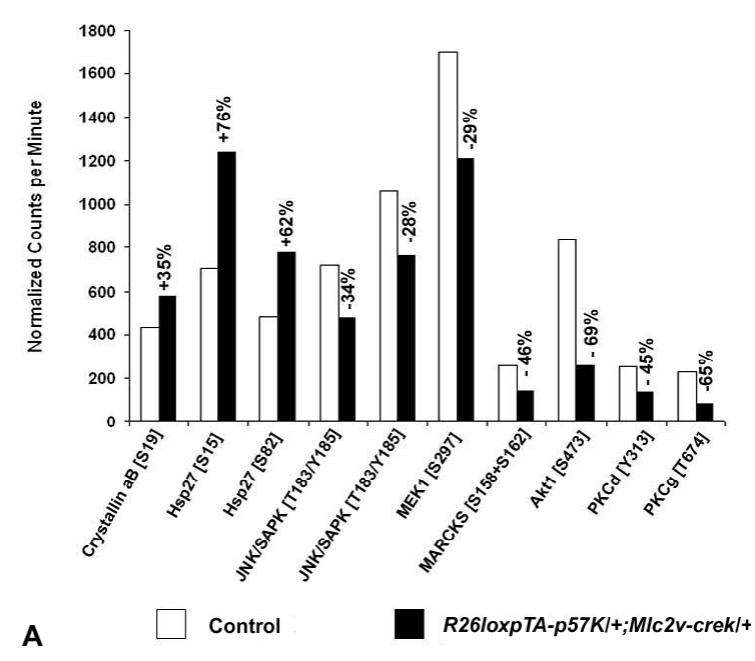
**p57^Kip2^-mediated cardioprotection in the *ex vivo *ischemia-reperfusion injury is associated with modulation of the stress signaling response**. Phospho-site screen analysis of key stress signaling molecules was performed by standardized densitometric quantification of Western immunoblots by *Kinexus*^*Tm *^Bioinformatics Corporation (Vancouver, Canada). The graph includes stress signaling molecules that display a significant difference between transgenic and control hearts.

Since p57^Kip2 ^has been found to physically interact with and inhibit JNK/SAPK, we investigated whether the cardioprotective effects of p57^Kip2 ^were mediated through modulation of the JNK stress signaling pathway. We performed a phospho-proteomic screen that demonstrated wide ranging changes in the stress signaling cascade. These included a 28–34% reduction in phosphorylation of the JNK/SAPK epitopes Y185 and T183 from ischemic p57^Kip2 ^overexpressing hearts compared to similarly ischemic control hearts after normalization to control for inaccuracies in protein determination and sample loading (Figure 6). In addition, phosphorylation changes in other stress signaling molecules not known to be directly related to p57^Kip2 ^such as αB-crystallin and heat shock protein 27 (Hsp27), MEK1, MARCKS, Akt1, PKCδ and PKCγ were also found in this screen.

## Discussion

p57^Kip2 ^encodes a maternally expressed cyclin dependent kinase inhibitor of the CIP/KIP family. While loss of function studies on p57^Kip2 ^have provided important functional clues, herein we report over-expression experiments that provide additional insight into its function. For better understanding of the role of p57^Kip2 ^in the cardiac tissue, we created a transgenic model that forces expression of p57^Kip2 ^beyond its narrow temporal window of expression. In our model, p57^Kip2 ^expression was observed from early gestation (E9.5) and the tissue-specific pattern persisted into adult life at 2.7 fold higher levels over the wild type controls. Cardiac specific expression of p57^Kip2^beginning at E9.5 did not appear to be deleterious, as *R26loxpTA-p57*^*k*/+^*;Mlc2v-Cre*^*k*/+ ^transgenic mice displayed normal cardiac growth both during development and postnatal life. This is in contrast to the observed effects of p57^Kip2 ^on other tissues, such as kidney and brain, that were shown to be sensitive to an even lower p57^Kip2 ^dosage change in BAC transgenic animals [29]. In addition, DNA synthesis persisted in the p57^Kip2 ^over-expressing cardiomyocytes, suggesting that the ability of cardiomyocytes to enter S-phase remained unimpaired. It is likely that sufficient amounts of the necessary CDKs are present in the fetal cardiomyocytes to overcome the induced excess of p57^Kip2^. Alternatively, excess p57^Kip2 ^could also have been removed by ubiquitination of the QT domain and degraded via the proteasome pathway [30,31]. However, the widely present p57^Kip2 ^protein in the fetal and adult cardiomyocytes, beyond the spatial and temporal pattern of endogenous expression, indicates that the cellular capacity for p57^Kip2 ^degradation was overcome and not sufficient to normalize the elevated protein levels. The concurrent expression of p57^Kip2 ^in actively proliferating cells is intriguing and suggests that, in cardiomyocytes, p57^Kip2 ^expression and terminal differentiation are not necessarily associated with cell cycle exit but rather with endoreduplication as in trophoblasts [24].

In our studies, we found that forced expression of p57^Kip2 ^in the adult heart was associated with a protective effect when the heart undergoes injury by transient ischemia/reperfusion. It seems unlikely that the cell cycle inhibitory function of p57^Kip2 ^is a factor in this process, as the protective effect was immediate. An alternative explanation to consider is the improved coronary flow in the transgenic hearts. Ischemia and reperfusion significantly increase tissue edema in the injured myocardium with secondary compromise of the regional coronary flow [32]. Since the p57^Kip2 ^transgene expression is restricted in cardiomyocytes and not present in smooth muscle or endothelial cells, we believe that the preservation of coronary flow is rather secondary to the decreased tissue edema in the transgenic hearts than a primary improvement in coronary vascular flow. Instead, it is more plausible that a separate function of p57^Kip2 ^leads to cardiac protection in the hypoxic phase of I/R injury. Our experiments support the possibility that p57^Kip2 ^modulates the stress signaling response after ischemia-reperfusion challenge. For example, we observed lower levels of JNK activity in the p57^Kip2 ^overexpressing mice. p57^Kip2 ^was shown to modulate stress-activated signaling and apoptosis by inhibiting c-Jun amino-terminal kinase/stress-activated protein kinase (JNK/SAPK) in cultured human embryonic kidney cells, embryonic fibroblasts and myoblasts [17]. JNK is activated following ischemia/reperfusion injury, and when the kinase activity is repressed, the area of infarct damage is diminished [33]. However, phosphorylation alterations were not restricted to the JNK/MAPK but involved other stress signaling molecules not known to be directly related to p57^Kip2 ^such as crystalline aB and heat shock protein 27 (Hsp27), MEK1, MARCKS, Akt1, PKCδ and PKCγ. While activation of some of these proteins is reported to be protective in cardiac disease (heat shock proteins), genetic and pharmacologic manipulation of some others such as JNK/MAPK, Akt1 and PKC has been reported to have conflicting or unknown roles, with both protective and detrimental ramifications for cardiomyocytes after *in vitro *and *in vivo *hypoxic injury [34-39]. Although, the mechanism for these phosphorylation changes is not known at this point, these data support the possibility that the p57^Kip2^-mediated cardioprotection is associated with wide changes of the stress signaling cascade. These findings indicate that p57^Kip2 ^protein may be a converging point for the regulation of cellular stress, cell proliferation and apoptosis.

Studies from human patients with both acute and end-stage heart failure reveal that CKIs revert to a fetal pattern of expression, i.e., p21^Cip1^and p27^Kip1 ^decline, while p57^Kip2 ^is significantly increased [16]. In addition, a recent analysis of the transcriptional regulation of the human proximal tubular epithelial cell response to hypoxia identified *p57*^*Kip*2 ^among a tightly regulated cluster of 48 genes that demonstrated time-dependent up-regulation in response to hypoxia and a distinct down-regulation upon reoxygenation [18]. Significantly, *p57*^*Kip*2 ^was up-regulated at early time points of hypoxia indicating it was within the early wave of hypoxia-responsive genes rather than being secondarily induced [18]. p57^Kip2 ^is normally highly expressed in the heart during midgestation, a time when the coronary arteries are not yet connected to the aortic root and the fetal heart grows in a low oxygen tension environment. These observations suggest a protective role for p57^Kip2 ^under conditions of limited oxygen supply. Our results provide additional support for this protective role of p57^Kip2 ^in the setting of hypoxia by demonstrating that persistent expression of p57^Kip2 ^in cardiomyocytes attenuates the ischemia-reperfusion injury in the adult mouse heart.

*p57*^*Kip*2 ^over-expression has been reported by us and other investigators in two independent mouse models of thin myocardium, one resulting from mutation of the *Pax3 *transcription factor (*Splotch*) [14] and the other from deletion of the secreted factor *Bmp10 *[40]. This phenotype was associated with a reduction in cardiomyocyte proliferative activity, while there was no evidence of increased apoptosis. These findings are consistent with early suppression of cardiomyocyte replication and increased differentiation associated with enhanced activity of *p57*^*Kip*2 ^in the mutant mouse hearts. The mechanism for *p57*^*Kip*2 ^up-regulation in these two models remains unknown, but given the different nature of their genetic defects, a direct transcriptional regulation is unlikely to be involved. An alternative explanation for this finding could be that *p57*^*Kip*2 ^up-regulation represents a secondary, adaptation type of response, or a selective survival of the p57^Kip2 ^expressing cardiomyocytes under conditions of increased stress imposed on the developmentally impaired thin myocardial wall.

## Conclusion

We have generated a transgenic mouse model that allows specific forced expression of *p57*^*Kip*2 ^in cardiomyocytes. The forced expression of p57^Kip2 ^in cardiomyocytes did not affect heart development, growth or baseline cardiac function. However, the continuous presence of p57^Kip2 ^in the adult mouse heart results in resistance to myocardial ischemia/reperfusion injury and improved recovery of cardiac function. Preservation of myocardiac function after ischemia/reperfusion depends on critical adaptive responses of the stress signaling network. In the case of *p57*^*Kip*2 ^overexpression, the exact mechanism of this cardioprotection has not been fully elucidated, but it was associated with wide range modulations of proteins in stress signaling pathways. It is conceivable that p57^Kip2 ^is part of the pre-conditioning process that protects the myocardium from ischemia-reperfusion injury and may constitute a new therapeutic target for ischemic cardiac disease.

As hypoxic stress contributes to many biological disorders, p57^Kip2 ^may be of general physiological importance for controlling cell proliferation and death under conditions of limited oxygen availability during embryonic cardiac development and pathologic ischemic conditions of adulthood. Comparison of the physiological state of embryonic development with pathologic conditions of cardiac stress in adulthood, suggests that in adult cardiomyocytes, the genetic response to stress is to revert to the expression of fetal genetic patterns associated with embryonic cardiac development. *p57*^*Kip*2 ^appears to be one of these fetal genes, which re-emerges under conditions of hypoxic/ischemic stress. Thus, the hypoxic developmental history may provide a useful insight in the adult heart's genetic response to ischemia, by the redeployment of genes that were adapted to the low oxygen tension characteristic of the fetal/embryonic environment.

## Methods

### Mice

A gene-targeting vector was constructed using a ROSA26 targeting vector (*pROSA26PA*) [41]. A 1.4 kb cDNA containing the complete open reading frame of mouse *p57*^*Kip*2 ^(generously provided by S. Elledge) was inserted into the *pBigT *vector [41] downstream to a loxP-flanked transcriptional termination sequence (tpA). This fragment was then inserted into the *pROSA26PA *plasmid [41,42] to create the targeting vector, now designated *R26loxp-TAp57 *(Figure 1). The *R26loxp-TAp57 *plasmid was electroporated into AB2.2 mouse embryonic stem (ES) cells and successful homologous recombination was determined by PCR analysis and confirmed by Southern blotting. The primers used were: *ROSA26FL *5'-CCTAAAGAAGAGGCTGTGCTTTGG*-3' *and *ROSA26SA 5'*-*CATCAAGGAAACCCTGGACTACTG-3'*.

Two separate *R26loxp-TAp57 *harboring ES clones were injected into C57BL/6-Tyr^c-Brd ^blastocysts and implanted into pseudo-pregnant females for the generation of chimeras. The male chimeras were mated with C57BL/6-Tyr^c-Brd ^to obtain F1 progeny. Eight animals with germline transmitted alleles were derived from two independently targeted ES cell lines. These mice were designated as *R26loxpTA-p57*^*k*/+^. *R26loxpTA-p57*^*k*/+ ^mice were bred with knock-in mice carrying the *Cre-recombinase *gene under control of the endogenous mouse *Mlc2v *promoter (generously provided by J. Chen, UCSD) to induce myocardial specific expression of *p57*^*Kip*2^. Mice were bred on a mixed C57BL/6 or 129/Sv background and were genotyped using tail extracted genomic DNA for diagnostic PCR amplification (Figure 1C).

Mice were evaluated by genomic DNA analysis at 3 weeks and at the time of their sacrifice (14–18 weeks). To detect the various alleles (*ROSA *wild-type:1.2 kb, transgenic: 6 Kb, or recombinant transgenic allele: 3.3 kb) we used the primers *ROSAFORW 5'-CTCCTCAGAGAGCCTCGGCTAGGTAGGGGATC*-3' and *ROSAREV 5'-GGGCGTTGTCCTGCAGGGGAATTGAACAGGTG-3*. The inserted *p57*^*Kip*2 ^was not expressed unless cre-mediated recombination removed the transcriptional termination sequence. A proofreading Taq polymerase for long transcripts (*LA Taq*™ DNA Polymerase, *TAKARA Mirus Inc*, Japan) was used with the following PCR protocol: 94°C for 1 min, followed by 30 cycles of 94°C for 30 sec and annealing/extension at 67°C for 6 min and 10 minutes extension at 72°C. The *cre *allele (300 bp) was detected by PCR using the primers *CREFORW 5'-GTTCGCAAGAACCTGATGGACA-3' *and *CREREV 5'-CTAGAGCCTGTTTTGCACGTTC-3'*.

Experiments were performed according to guidelines from the National Institutes of Health and with an approved protocol from the Animal Care and Use Committee.

### Histological analysis

Mouse tissues and embryos were fixed with 2% PFA overnight, dehydrated, embedded in paraffin, and sectioned at 5 μm thickness. Primary antibodies included anti-p57^Kip2 ^(Ab-4058, Abcam inc. Cambridge, MA; H-91, Santa Cruz BioTechnologies, Santa Cruz, CA) at 1:50, Ki-67 (550609, BD Pharmingen, San Jose, CA) at 1:50, and anti-MHC (MF-20, from DHSB, University of Iowa) at 1:150. Secondary detection occurred by fluorescence with the anti-rabbit Cy3 (Jackson Immunoresearch, West Grove, PA) or anti-mouse Alexa 488 (Molecular Probes, Eugene, OR) at 1:200 and mounted in media containing DAPI (Vectashield, Vector Laboratories, Burlingame, CA). For immunohistochemistry, a biotinylated anti-rabbit antibody was used at 1:200 and developed using the ABC-DAB system (Vector Laboratories). Slides were counterstained with Methylene Blue and mounted in Permount.

### RT-PCR

Total RNA was isolated from control and mutant adult hearts by Trizol (Invitrogen, Carlsbad, CA) extraction. The PCR reactions were performed with the 7500 RT-PCR system (Applied Biosystems, Foster City, CA) using Brilliant SYBR Green QRT-PCR Master Mix (Stratagene, La Jolla, CA) and the housekeeping gene β-actin for internal normalization. All samples were tested in triplicates for each genotype and the results represent averages of individual experiments.

### Langendorff isolated perfused heart and assessment of cardiac function

Adult male mice (14–30 weeks) were euthanized with an intraperitoneal injection of pentobarbital sodium. Hearts were rapidly excised and arrested in ice-cold Krebs-Henseleit buffer, which was constantly gassed with 95% O_2 _and 5% CO_2 _to give a pH of 7.4 at 37°C. The buffer was perfused at a constant pressure of 55 mm Hg in the non-recirculating Langendorff mode as previously described [28]. Briefly, the hearts were cannulated via the ascending aorta for retrograde perfusion. A left atrial incision was made to expose the mitral annulus, through which a water-filled latex balloon was passed into the LV. The balloon was attached to a pressure transducer, which was connected to an A/D Converter. The converter transmits the data to a computer running a cardiovascular data acquisition software and recording system (Biopac, MP 100). The LV balloon was inflated to adjust the LV end-diastolic pressure (LVEDP) to ≈ 10 mm Hg. Myocardial function was measured at multiple, independent end points. The following functional parameters were monitored and constantly recorded: left ventricular developed pressure (LVDP), which is the difference of left ventricular end systolic pressure (LVESP) minus left ventricular end-diastolic pressure (LVEDP), heart rate, coronary flow (CF), the maximum positive or negative first derivative of left ventricular pressure (± dP/dt_max/min_) (as index of inotropic and relaxation state respectively), and the rate pressure product (RPP=LVDPXHR) was calculated and used as index of cardiac work and indirect measure of myocardial oxygen utilization (VO_2_).

### Protein extraction and phospho-proteomic screen

Hearts (two from wild type and two from transgenic mice) were collected after baseline evaluation or after ischemia-reperfusion *ex-vivo *injury, snap-frozen in liquid N_2 _and stored at -80°C. The frozen hearts were washed with ice-cold PBS and cut into small pieces in protein lysis buffer containing 0.5% Triton X-100 and proteinase inhibitors. Cardiac tissue lysates were prepared by homogenization and sonication with a Brickmann Polytron homogenizer and a Fisher Sonic Dismembrator. Protein concentrations were determined with the bicinchoninic acid assay (BCA™ Protein Assay kit, Pierce, Rockford, IL). The phospho-proteomic screen was performed and analyzed by Kinexus Inc. (Vancouver, Canada).

### Statistical analysis

For parameters that require quantification and evaluation for statistical significance, results were expressed as mean ± standard error of the mean (SEM). Statistical significance (probability values) was determined using the student's t-test (two tailed distribution and two sample unequal variance). For multiple group comparisons, one way analysis of variance (ANOVA) followed by *Fisher post hoc *test was used. A probability of p = 0.05 was considered to represent statistical significance. Differences in observed *vs *expected numbers of a particular genotype were determined using the Chi-squared test.

## Abbreviations

CDK, cyclin dependent kinase; CKI, cyclin dependent kinase inhibitor; CF, coronary flow; RPP, rate pressure product; LVDP, left ventricular developed pressure; LVEDP, left ventricular end-diastolic pressure; LVESP, left ventricular end systolic pressure

## Authors' contributions

SAH: created the targeting construct, performed the molecular studies and drafted the manuscript.

TZ: performed the cardiac function experiments with the Langendorff isolated perfused heart system and analyzed their results.

LJ: extracted protein and performed immunoassays, managed the animal colony including genotyping, and coordinated the experimental progress.

JAK: performed and supervised the mouse ES cell work and the generation of the transgenic mice as Director of the Animal Transgenic Facility at Brown University.

JFP: participated in the design and coordination of the study and critically reviewed the manuscript.

LKK: conceived and designed the study, interpreted the results, coordinated the experiments, helped to draft the manuscript and critically reviewed and revised its final version.

All authors read and approved the final manuscript.
